# Dietary Energy Density in Relation to Subsequent Changes of Weight and Waist Circumference in European Men and Women

**DOI:** 10.1371/journal.pone.0005339

**Published:** 2009-04-27

**Authors:** Huaidong Du, Daphne L. van der A, Vanessa Ginder, Susan A. Jebb, Nita G. Forouhi, Nicholas J. Wareham, Jytte Halkjær, Anne Tjønneland, Kim Overvad, Marianne Uhre Jakobsen, Brian Buijsse, Annika Steffen, Domenico Palli, Giovanna Masala, Wim H. M. Saris, Thorkild I. A. Sørensen, Edith J. M. Feskens

**Affiliations:** 1 National Institute for Public Health and the Environment (RIVM), Bilthoven, The Netherlands; 2 Department of Human Biology, Nutrition and Toxicology Research Institute of Maastricht (NUTRIM), Maastricht University, Maastricht, The Netherlands; 3 Division of Human Nutrition, Wageningen University, Wageningen, The Netherlands; 4 Nutrition and Health Research, MRC Human Nutrition Research, Cambridge, United Kingdom; 5 MRC Epidemiology Unit, Institute of Metabolic Science, Cambridge, United Kingdom; 6 Danish Cancer Society, Institute of Cancer Epidemiology, Copenhagen, Denmark; 7 Department of Cardiology, Aalborg Hospital, Aarhus University Hospital, Aalborg, Denmark; 8 Department of Clinical Epidemiology, Aarhus University Hospital, Aalborg, Denmark; 9 Department of Epidemiology, German Institute of Human Nutrition, Potsdam, Germany; 10 Molecular and Nutritional Epidemiology Unit, Cancer Research and Prevention Institute (ISPO), Florence, Italy; 11 Institute of Preventive Medicine, University Hospital, Centre for Health and Society, Copenhagen, Denmark; UCL Institute of Child Health, United Kingdom

## Abstract

**Background:**

Experimental studies show that a reduction in dietary energy density (ED) is associated with reduced energy intake and body weight. However, few observational studies have investigated the role of ED on long-term weight and waist circumference change.

**Methods and Principal Findings:**

This population-based prospective cohort study included 89,432 participants from five European countries with mean age 53 years (range: 20–78 years) at baseline and were followed for an average of 6.5 years (range: 1.9–12.5 years). Participants were free of cancer, cardiovascular diseases and diabetes at baseline. ED was calculated as the energy intake (kcal) from foods divided by the weight (g) of foods. Multiple linear regression analyses were performed to investigate the associations of ED with annual weight and waist circumference change.

Mean ED was 1.7 kcal/g and differed across study centers. After adjusting for baseline anthropometrics, demographic and lifestyle factors, follow-up duration and energy from beverages, ED was not associated with weight change, but significantly associated with waist circumference change overall. For 1 kcal/g ED, the annual weight change was −42 g/year [95% confidence interval (CI): −112, 28] and annual waist circumference change was 0.09 cm/year [95% CI: 0.01, 0.18]. In participants with baseline BMI<25 kg/m^2^, 1 kcal/g ED was associated with a waist circumference change of 0.17 cm/year [95% CI: 0.09, 0.25].

**Conclusion:**

Our results suggest that lower ED diets do not prevent weight gain but have a weak yet potentially beneficial effect on the prevention of abdominal obesity as measured by waist circumference.

## Introduction

The global obesity epidemic triggers research investigating the dietary determinants of a positive energy balance. The energy density (ED) of foods or diets, defined as the amount of available energy per unit weight of foods or meals (kJ/g or kcal/g)[1], has gained much attention in this respect[2]. For example, the joint WHO / FAO expert consultation on diet, nutrition and the prevention of chronic diseases classifies the evidence of a positive relationship between high intake of energy-dense foods and weight gain and obesity as convincing[3]. In addition, the Dietary Guidelines for Americans 2005 recommend eating foods that are low in calories for a given measure of food to reduce calorie intake[4].

Experimental data suggest that people tend to eat a similar volume of food to feel satiated, and, accordingly, consuming energy-dense foods could cause passive over-eating in terms of energy[2]. Furthermore, energy-dense foods high in fat and sugar tend to be highly palatable and stimulate over-eating[5]. Some cross-sectional studies show a positive association between ED and obesity but there are concerns about reverse causality[1], [6]. Intervention studies among overweight and obese subjects consistently demonstrate that ED reduction is associated with weight loss[7], [8], [9]. However, these interventions were of relatively short period. Findings from observational studies, on the other hand, are less consistent. For instance, in two recently published studies among US women, higher ED has been found to predict higher weight gain[10], [11]. However, no such significant relationship was observed in an earlier study among Danish adults[12]. In a study among British children, higher ED at seven years of age, but not at 5 years of age, has been found to be a risk factor for excessive adiposity at the age of nine years[13].

The present study was conducted to examine the prospective relationship between dietary ED and long-term (1.9–12.5 years) changes in body weight and waist circumference within a large European study, which is a part of the DiOGenes project (acronym for “**Di**et, **O**besity and **Genes**”)[14].

## Methods

### Participants

The current study included participants from eight cities or counties in five different countries involved in the European Prospective Investigation into Cancer and Nutrition (EPIC) study, namely Florence (Italy), Norfolk (the UK), Amsterdam, Maastricht and Doetinchem (The Netherlands), Potsdam (Germany), Copenhagen and Aarhus (Denmark). EPIC study has been approved by local review board of all participating institutions. Written informed consent has been obtained from all participants before joining EPIC study. Detailed information on the study population and data collection of the EPIC study has been described elsewhere[15]. Of the 146,543 participants who took part in the baseline examination during 1992–1998, 102,346 (69.8%) participated in the follow-up examination during 1998–2005. For the present study, the following exclusion criteria were applied: pregnancy (n = 133), missing information on diet, anthropometrics or follow-up time (n = 2,135), the ratio of energy intake (EI) to estimated basal metabolic rate (BMR) (EI: BMR) being top or bottom 1% of the entire EPIC population (n = 1,803), unrealistic anthropometric measurements (n = 331) and those with history of cancer, diabetes or cardiovascular diseases (CVD) at baseline (n = 8,512). In total, 89,432 participants, 37,125 (42%) men and 52,307 (58%) women, were included in the analyses.

### Dietary assessment

Country-specific food frequency questionnaires (FFQs) were self-administered at baseline. Energy and nutrient intakes were calculated using country-specific food composition tables[15]. ED was calculated as energy (kcal) from foods (solid foods and semi-solid or liquid foods such as soups) divided by the weights (g) of these foods. Drinks (including water, tea, coffee, juice, soft drinks, alcoholic drinks and milk) were not included in the calculation[16]. To improve comparability of dietary data collected using different FFQs, and to adjust for measurement errors, linear calibration was performed with single 24-hour dietary recall from a stratified random sample of the total EPIC study population as reference method[17], [18]. Among the 89,432 participants included in the current study, 6,790 participants had also 24-hour dietary recall data available. This 24-hour dietary recall was collected using a software program (EPIC-SOFT) specifically designed to standardize the dietary measurements across European populations[19]. Nutrient intake in this 24-hour recall data were standardized based on the standardized nutrient database developed within the EPIC study (ENDB)[20]. Gender- and center-specific calibration models were built with 24-hour recall data as the dependent variable and FFQ data as the independent variable. Age, weight, height, and season in which the FFQ measurement was conducted were adjusted for. Under-reporting of EI was assessed by EI: BMR<1.1, [11] where BMR was estimated using Harris-Benedict equations[21].

### Anthropometric measurements

Weight and waist circumference were collected at baseline and at the end of follow-up. At baseline, all participants were measured by trained technicians for weight, height and waist circumference using standard study protocols as previously described[22]. At follow-up, participants in Doetinchem (NL) and Norfolk (UK) were measured by trained technicians, while those in the other centers provided self-reported weight and waist circumference. Annual changes in weight (g/year) and waist circumference (cm/year) were calculated as follow-up values minus baseline values and divided by the total years of follow-up. Due to differences in methods used to collect anthropometric information at follow-up and the length of follow-up time, participants from Doetinchem (NL) were treated separately from those from Amsterdam and Maastricht (NL), while participants from Copenhagen and Aarhus (DK) were combined because no such differences between these two groups existed. Thus six study centers were formed, namely Florence (IT-Flo) (n = 9,297, 10%), Norfolk (UK-Nor) (n = 12,808, 14%), Amsterdam-Maastricht (NL-AmMa) (n = 6,911, 8%), Doetinchem (NL-Doe) (n = 4,200, 5%), Potsdam (GER-Pot) (n = 16,307,18%) and Copenhagen-Aarhus (DK-CopAa) (n = 39,909, 45%).

### Other covariates

Information on lifestyle was collected via self-administrated questionnaires. Questions covered demography (age, gender), education level, physical activity, smoking, menopausal status, and use of hormone replacement therapy (HRT). Information on health status, including CVD, cancer and diabetes, was collected using either questionnaires or disease registries. Assessment of physical activity level was indexed into four categories based on their occupational and recreational activities. Education level was inquired as the highest level of school achieved. Based on the information of smoking status at baseline as well as at follow-up, participants were classified into one of the following four categories: stable smoking, start smoking, quit smoking, non- smoking or unknown.

### Statistical methods

Characteristics of participants were given along quintiles of ED. Stepwise regression analyses were conducted to investigate the contribution of food groups and nutrients to the inter-individual variation in ED. Fifteen food group variables were entered into the regression model, including potatoes, vegetables, legumes, fruits, dairy products, cereals, meat, fish, eggs, fats, sugar and confectionery, cakes, condiments and sauces, soups and miscellaneous[23]. As for nutrients, six macronutrient variables, including saturated fatty acids, monounsaturated fatty acids, polyunsaturated fatty acids, polysaccharides, mono-&disaccharides and protein were entered in the regression model.

The association of ED with annual weight and waist circumference change was investigated using multiple linear regression analyses. Center-specific analyses were first performed and random-effect meta-analyses were used to evaluate heterogeneity (*I^2^*) and obtain pooled estimates of associations. Analyses were adjusted for a pre-decided set of potential confounders including baseline age (years), gender, height (cm), baseline weight (kg) and waist circumference (cm, only for waist circumference change analyses), smoking, physical activity (inactive, moderately inactive, moderately active, active or missing), education (primary school and less, technical-professional school, secondary school, university degree or missing), follow-up time (years), alcohol intake (non-drinker, 0.1–4.9 g/day, 4.9–15 g/day, 15–30 g/day, 30–60 g/day, >60 g/day), energy intake from beverages, and among women only, menopausal status (postmenopausal yes or no) and HRT use (yes, no, or missing).

Interactions of ED with age, gender, baseline BMI, smoking, EI:BMR, follow-up duration, and baseline waist circumference (for waist circumference change analyses only) were investigated by introducing product terms into the models. A two-sided *P*<0.05 was considered statistically significant in the analyses of main effects, whereas *P*<0.01 in at least three of the six study centers was considered relevant when testing of interaction.

We performed several sensitivity analyses, including use un-calibrated dietary variables; use corrected anthropometric variables based on the equations developed in EPIC study[24]; exclude participants who developed type 2 diabetes, cancer, or CVD during follow-up; and additionally adjust for individual food or nutrient variables which potentially mediate the effects of ED on weight and waist circumference change, including total energy, fruits, vegetables, total fat, saturated fatty acids, dietary fiber, glycemic index and glycemic load. Except for the random-effect meta-analyses, which were conducted using STATA 8.2 (StataCorp, Texas, USA), all other statistical analyses were performed using SAS 9.1 (SAS, Institute, Cary, NC).

## Results

The mean baseline age was 53 years (range: 20–78 years) and mean follow-up duration was 6.5 years (range: 1.9–12.5 years). At baseline, 12% of participants were obese (BMI≥30 kg/m^2^); a further 41% were overweight (30 kg/m^2^>BMI≥25 kg/m^2^); and 21% had abdominal obesity (waist circumference ≥102 cm for men and ≥88 cm for women) (data not shown). On average, participants in NL-AmMa were the youngest at baseline (42 years) and were followed for a longest period (10 years), while those from UK-Nor were the oldest at baseline (58 years) and with the shortest follow-up duration (3.7 years). Annual weight change was higher in NL-Doe (mean = 440 g/year) and UK-Nor (374 g/year) compared to the weight change in the other centers with self-reported weight at follow-up (164 g/year, 164 g/year, 183 g/year and −51 g/year respectively for participants from IT-Flo, NL-AmMa, GER-Pot and DK-CopAa). However, for waist circumference change, the highest value was observed in DK-CopAa (0.96 cm/year), followed by the waist circumference change in the IT-Flo (0.84 cm/year), GER-Pot (0.76 cm/year), NL-AmMa (0.63 cm/year), and NL-Doe (0.58 cm/year), and the lowest in UK-Nor (0.22 cm/year).

The overall mean ED was 1.7 kcal/g and higher in men (1.9 kcal/g) than in women (1.6 kcal/g). Participants in NL-AmMa had the highest ED (1.9 kcal/g), followed by those from DK-CopAa (1.8 kcal/g), UK-Nor (1.7 kcal/g), NL-Doe (1.7 kcal/g), and GER-Pot (1.6 kcal/g), and the lowest was observed among those from IT-Flo (1.5 kcal/g). Although consuming lower amount (total grams) of foods, participants with higher ED had greater intake of total energy and energy from beverages. They smoked more and were more physically active. Those in the higher ED quintile groups also had lower fiber intake and higher dietary glycemic index and glycemic load **(** **TABLE 1** **)**.

**Table 1:** Characteristics of the study population across quintiles of dietary energy density (n = 89,432).

Characteristics*	Overall	Q1	Q2	Q3	Q4	Q5
Energy density, kcal/g	1.7±0.27	1.4	1.6	1.7	1.9	2.1
Baseline age, *yrs*	53±8.6	54	54	54	53	52
Gender, % of men	42	11	26	41	58	73
Follow-up duration, *yrs*	6.5±2.2	7.3	6.6	6.2	6.1	6.2
Baseline weight, *kg*	73.4±13.5	69.4	71.0	73.2	75.7	77.8
Baseline BMI, *kg/m^2^*	25.7±3.8	25.9	25.7	25.6	25.7	25.7
Baseline waist circumference, *cm*	86±12	82	84	86	88	90
Total energy, *kcal/day*	2,200±460	1,860	2,032	2,197	2,363	2,549
Energy from beverages, *kcal/day*	350±169	260	302	348	394	447
Total gram of foods, *g/day*	1,308±260	1,315	1,312	1,318	1,316	1,281
Fiber†, *g/day*	22.8±4.0	23.6	22.9	22.9	22.8	22.1
Glycemic index†	57±2.0	55	56	57	58	59
Glycemic load†	134±22	124	130	134	139	143
Smoking status‡, %						
Stable smoking	19	12	14	17	22	31
Start smoking	2	2	1	2	2	2
Quit smoking	7	6	6	6	7	9
Non-smoking	72	81	79	75	69	58
Education§, %						
Primary school or lower	27	30	28	25	25	28
Technical/professional school	36	33	36	37	38	37
Secondary school	13	15	14	13	12	11
University degree or higher	23	22	22	24	26	24
Physical activity∥, %						
Inactive	16	19	18	16	14	13
Moderately inactive	33	36	35	33	32	29
Moderately active	24	23	23	25	25	24
Active	27	22	24	26	29	34
Menopausal status¶, % post-menopausal	57	58	57	57	56	57
Hormone replacement therapy¶ ^,^ #, % users	22	21	22	22	22	22

* Expressed as means (or mean ± SD), otherwise indicated. Differences between quintile groups were tested using chi-square test (categorical variables) or ANOVA test (continuous variable). *P*<0.0001 for all.

† Energy-adjusted residuals of dietary variables.

# 1,273 participants with missing values.

‡ 1,440 participants with missing values.

§ 1,579 participants with missing values.

∥ 3,319 participants with missing values.

¶ for women only.

Percentages are based on those participants with available data on that variable and may not sum to 100% due to rounding.

Among foods, fruits explained the most variation in ED (35%), followed by sugar and confectionery, fats, and vegetables (13%, 8% and 7% respectively) (**TABLE 2**). ED was inversely associated with the intake of fruits and vegetables but positively associated with the intake of fats and sugar and confectionery. Among nutrients, saturated fatty acids explained the most variation in ED (24%).

**Table 2:** Relationships of food groups and macronutrients with dietary energy density (kcal/g) (n = 89,432).

Food groups	β*	Partial R^2^ †	Model R^2^
Fruits	−0.10	0.35	0.35
Sugar and confectionery	0.32	0.13	0.48
Fats	0.46	0.08	0.56
Vegetables	−0.19	0.07	0.63
Cereals and cereal products	0.07	0.06	0.69
Soups and bouillon	−0.19	0.04	0.73
Potatoes and other tubers	−0.10	0.02	0.75
Cakes and cookies	0.20	0.01	0.76
Meat and meat products	0.10	0.01	0.77

Macronutrients	β‡	Partial R^2^	Model R^2^
Saturated fatty acids	0.04	0.24	0.24
Mono- & disaccharides	−0.02	0.08	0.32
Protein	−0.04	0.05	0.37

* β regression coefficients refer to the energy density (kcal/g) difference explained by 100 g foods.

† Only food or nutrient items had Partial R^2^>0.01 were listed here.

‡ β regression coefficients refer to the energy density (kcal/g) difference explained by 1% of energy contributed by individual nutrient.

**Figure 1:**
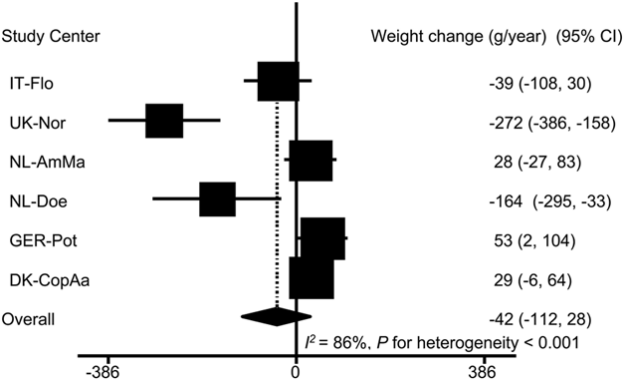
Association of energy density with annual weight change (n = 89,432)^*^. 95% CI: 95% confidence interval of regression coefficients. Regression coefficients represent the annual weight change (g/year) for 1 kcal/g ED. The overall estimate was based on random-effect model. ^*^ Adjusted for follow-up time and baseline age, height and weight, smoking, physical activity, education, alcohol intake, menopausal status, hormone replace therapy use, and energy intake from beverages.

After adjusting for the aforementioned covariates, there was no significant overall association between ED and annual weight change. The mean weight change for each 1 kcal/g ED was −42 g/year [95% confidence interval (CI): −112, 28]. A significant degree of heterogeneity across study centers was observed (*I^2^* = 86%, *P* for heterogeneity <0.001). In IT-Flo, ED was inversely, but not significantly, associated with weight change. In NL-AmMa and DK-CopAa, the associations were toward a positive direction. The inverse associations in UK-Nor and NL-Doe and the positive association in GER-Pot were statistically significant (**FIGURE 1**).

**Figure 2:**
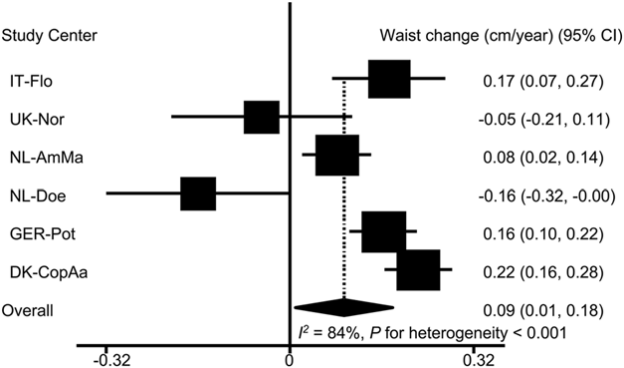
Association of energy density with annual waist circumference change (n = 89,432)^*^. 95% CI: 95% confidence interval of regression coefficients. Regression coefficients represent the annual waist circumference change (cm/year) for 1 kcal/g ED. The overall estimate was based on random-effect model. ^*^ Adjusted for follow-up time and baseline age, height, weight, and waist circumference, smoking, physical activity, education, alcohol intake, menopausal status, hormone replace therapy use, and energy intake from beverages.

ED was positively and significantly associated with waist circumference change in IT-Flo, NL-AmMa, GER-Pot and DK-CopAa. In UK-Nor, ED was inversely but not significantly associated with waist circumference change. In NL-Doe, ED was inversely and significantly associated with waist circumference change. Overall, 1 kcal/g ED was associated with a waist circumference change of 0.09 cm/year [95% CI: 0.01, 0.18] (*I^2^* = 84 %, *P* for heterogeneity <0.001) (**FIGURE 2**).

**Figure 3:**
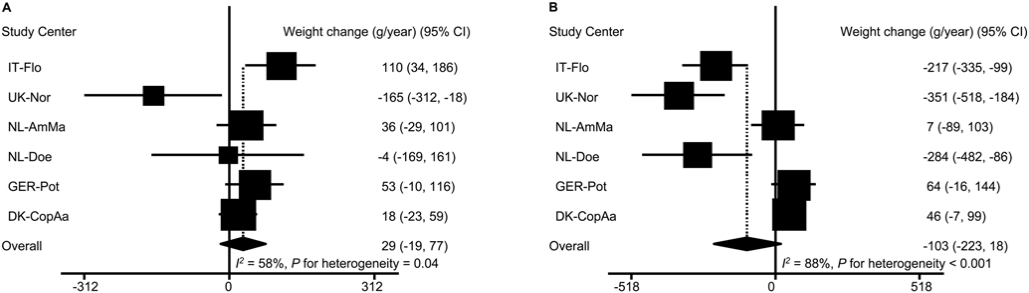
Association of energy density with annual weight change by baseline BMI^*^. A: for participants with BMI<25 kg/m^2^ (n = 41,914). B: for participants with baseline BMI≥25 kg/m^2^ (n = 47,518). 95% CI: 95% confidence interval of regression coefficients. Regression coefficients represent the annual weight change (g/year) for 1 kcal/g ED. The overall estimate was based on random-effect model. ^*^ Adjusted for follow-up time and baseline age, height and weight, smoking, physical activity, education, alcohol intake, menopausal status, hormone replace therapy use, and energy intake from beverages.

**Figure 4:**
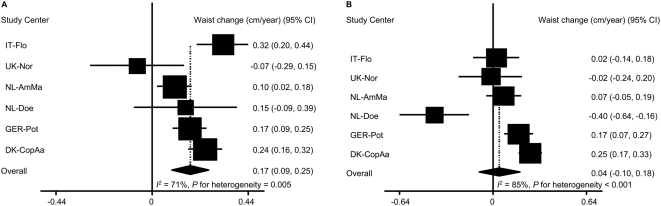
Association of energy density with annual waist circumference change by baseline BMI^*^.

Only baseline BMI fulfilled the pre-decided criteria for being a significant effect modifier. Among participants with baseline BMI<25 kg/m^2^, ED was in the direction of positively associated with weight change: 1 kcal/g ED was associated with a weight change of 29 g/year [95% CI: −19, 77] (**FIGURE 3A**). However, among those participants who were overweight or obese at baseline (BMI≥25 kg/m^2^), ED was inversely associated with weight change: 1 kcal/g ED was associated with a weight change of −103 g/year [95% CI: −223,18] (**FIGURE 3B**). In IT-Flo, ED was significantly and positively associated with weight change among those participants with BMI<25 kg/m^2^ but inversely among those BMI≥25 kg/m^2^. In UK-Nor and NL-Doe, the inverse association between ED and weight change was much weaker among participants who had a healthy BMI at baseline compared to the association in those who were overweight or obese. The differences in the other study centers were less evident. For the associations with waist circumference change, the most evident differences were observed in IT-Flo and NL-Doe. The associations in participants with BMI<25 kg/m^2^ were much stronger, in the positive direction, than the associations in those who were overweight or obese. Overall, 1 kcal/g ED was associated with a waist circumference change of 0.17 cm/year [95% CI: 0.09, 0.25] and 0.04 cm/year [95% CI: −0.10, 0.18] respectively in participants with BMI<25 kg/m^2^ (**FIGURE 4A**) and those with BMI≥25 kg/m^2^ (**FIGURE 4B**).

Associations remained essentially similar in all sensitivity analyses performed, including use un-calibrated dietary variables; use corrected anthropometric variables; exclude participants who developed type 2 diabetes, cancer, or CVD during follow-up; and additionally adjust for individual food or nutrient variables which potentially mediate the effects of ED on weight and waist circumference change (results not shown).

## Discussion

In this large multi-center prospective cohort study, we observed that a diet with a lower ED was associated with a lower intake of sugar and fats and a higher intake of fruits and vegetables. Higher ED was not associated with weight change but was positively associated with waist circumference change.

The main strengths of our study are the large sample size and variation in dietary intake based on participants from five European countries, and the availability of information on important confounding variables and predictors of weight or waist circumference change such as physical activity, education level, changes in smoking status, menopausal status and HRT use. Some differences in methodologies used to collect anthropometric data at follow-up (weight and waist circumference were self-reported instead of measured in four out of six centers) might have affected the results. However, in additional analyses, we corrected anthropometrics for clothing differences and self-reporting using previously developed methods in the EPIC study[24], but the associations of ED with changes in weight and waist circumference remained unchanged. We therefore opted to use the original uncorrected data in our analyses. Using FFQs to assess ED might be another limitation of the current study because FFQs, based on self-report of habitual food intake, may have inherent measurement error as well as bias from conscious or sub-conscious under-reporting or possible overreporting of some food items. We addressed this in two ways. First, we used standardized 24-hour recall data to calibrate the FFQ measurements, thereby reducing potential measurement error. In addition, we also compared ED measured by the FFQ used in the two Dutch centers (NL-AmMa and NL-Doe) and the ED derived from the weighted average of multiple 24-hour recalls in a preliminary study. Spearman correlation coefficient was 0.64 in men and 0.56 in women (unpublished data), which indicated a good validity of the ED values measured by this FFQ[25], [26].

No clear consensus has been reached yet on the calculation of ED. A previous review of the literature identified 13 different calculation methods which mostly differed in the inclusion or exclusion of drinks, including free water, alcoholic beverages, energy-containing beverages, juice and milk[12], [16], [27]. In the present study, we *a priori* decided to use the calculation method based on foods only but not drinks[28]. Beverages add more weight than energy to diets, thereby lowering ED disproportionately. Furthermore, energy from drinks has only transient effects on satiation and does not influence habitual energy intake[29]. Also, beverage intake is highly variable and difficult to be estimated by any habitual diet assessment method. Previous studies indicated that including drinks into the ED calculation would dilute the associations of ED with both energy intake and changes of weight and waist circumference[16], [30]. When comparing the mean ED of our study population with that of other populations calculated using a similar method, the average ED in the current study lies between the population average of Japan (1.4 kcal/g) and the US (1.9 kcal/g in men and 1.8 kcal/g in women)[6], [28].

The relationships of ED with energy, food and nutrient intake found in the current study are in agreement with previous findings[1], [13], [31], [32]. This may imply a high quality profile of diets with a lower ED because diets lower in glycemic index and glycemic load, containing higher amounts of fruits, vegetables and fiber and lower amounts of sugar and fats, especially saturated fatty acids, are generally believed to be favorable for promoting human health[4], [33], [34], [35]. It also provides a way for reducing ED, by consuming more fruits and vegetables and reducing sugar and fat consumption.

The mechanism linking higher ED diet consumption and large gain in weight and waist circumference was speculated through increasing total energy intake. Therefore, we did not adjusted for total energy in our main analyses. However, when energy intake was added in the models in additional analyses, the association of ED with weight and waist circumference change was not essentially changed. This indicates that other aspects than lowering energy intake, such as reducing fat storage, is responsible for the observed effects. Furthermore, additional adjustment for individual nutrient factor such as total fat, saturated fatty acids, fiber, glycemic index or glycemic load did not alter the associations either. This may suggest that lower ED reflects a healthy dietary pattern rather than any individual dietary component.

The absence of an association between ED and weight change in our current study are at variance with other prospective cohort studies that observed a positive association between ED and weight change. For example, in a longitudinal study of 186 women in the US, women with higher ED gained more weight than women with lower ED (6.4 kg vs. 2.5 kg over 6 years)[10]. In two prospective studies among British children, a positive association between ED and body fat mass gain has been observed[13], [30]. In the Nurses' Health Study II, women who increased their dietary ED during follow-up the most (5th quintile) had a significantly greater weight gain than did those who decreased their dietary ED (1st quintile) (6.42 kg vs. 4.57 kg over 8 years). However, weight gain was not different between women who maintained a lower ED and women who maintained a higher ED during follow-up[11]. This latter non-significant finding was in accordance with the finding in the MONICA study[12] and our current study. As explained by the authors, participants with a constantly higher ED might compensate for the energy intake from higher ED diet. The weight gain for those participants may have already reached a steady state after long-term consumption of a higher ED diet[11]. However, since the habitual diets of participants have only been measured once at baseline, it impossible to clarify this issue in the current study. Another difference between the current study and the previous studies is the wide age range of participants (20–78 years at baseline). Despite the fact that no interaction with age was found, it is possible that the speed of weight gain slowed down in older participants.

This prospective cohort study showed a positive overall association between ED and waist circumference change, which was not addressed in the abovementioned studies[10], [11], [12], [13]. Although the association was rather weak, a waist circumference change of 0.09 cm/year represents approximately 12% of the mean waist circumference change in this study population. Abdominal obesity, measured by waist circumference, is a more accurate predictor of cardio-metabolic risk than general obesity measured by BMI, probably because it more closely reflects body fatness[36]. The large variation in the annual waist circumference change (SD = 1.26 cm/year) may be partly responsible for the small magnitude observed. Because of the small average waist circumference change (0.76 cm/year) as compared to the large variation, it is impossible to detect a stronger association[37]. The weak association between ED and waist circumference, and the absence of an association between ED and weight change, could also be due to selective underreporting, which means underreporting of unhealthy foods and overreporting of healthy ones[38]. This is a common problem in epidemiological studies and compromises the accuracy of habitual dietary intake data, especially among overweight or obese individuals[39]. This is in line with our findings that the ED was in the direction of positively associated with weight change in those participants with a healthy BMI at baseline, whereas was inversely in overweight or obese participants. Furthermore, there is evidence that underreporting is less waist-related than weight-related[40], which may be due to the higher awareness of the importance of a healthy BMI than a healthy waist circumference. Therefore the association between ED and waist circumference change may be less biased by the underreporting.

The high amount of heterogeneity observed in the current study, especially the unexpected inverse association between ED and changes in weight and waist circumference in UK-Nor and NL-Doe where the follow-up anthropometrics were measured, may also be related to underreporting. That is because we observed a smaller heterogeneity across study centers in those participants who had a healthy baseline BMI than in those participants who were overweight or obese at baseline. More importantly, the inverse associations between ED and changes in weight and waist circumference in UK-Nor and NL-Doe became less inverse or positive in participants who were not overweight at baseline. The heterogeneity could also be due to the differences in source population between study centers. For example, in IT-Flo, participants include a sample of the general population and women participating in a breast cancer screening program. The UK-Nor cohort comes from general practitioners in the Norfolk region[41]. In NL-Doe, respondents from a pre-existing cardiovascular disease risk factor monitoring project were invited[42]. Although in general there is no reason to assume a priori that these cohorts are different with respect to the association under study, subtle differences in underreporting of dietary intake, possibility of altering eating habits during follow-up, and health conscious among sub-groups may exist.

In conclusion, the findings of the current study suggests that diets with a lower ED, characterized by higher intake of fruits and vegetables and lower intake of sugar and fat, are not associated with weight gain but may have a beneficial, albeit weak, effect on the prevention of abdominal obesity.
